# Rare coding variants pinpoint genes that control human hematological traits

**DOI:** 10.1371/journal.pgen.1006925

**Published:** 2017-08-07

**Authors:** Abdou Mousas, Georgios Ntritsos, Ming-Huei Chen, Ci Song, Jennifer E. Huffman, Ioanna Tzoulaki, Paul Elliott, Bruce M. Psaty, Blood-Cell Consortium, Paul L. Auer, Andrew D. Johnson, Evangelos Evangelou, Guillaume Lettre, Alexander P. Reiner

**Affiliations:** 1 Department of Medicine, Université de Montréal, Montréal, Québec, Canada; 2 Montreal Heart Institute, Montréal, Québec, Canada; 3 Department of Hygiene and Epidemiology, University of Ioannina Medical School, Ioannina, Greece; 4 Population Sciences Branch, National Heart Lung and Blood Institute, The Framingham Heart Study, Framingham, MA, United States of America; 5 Department of Epidemiology and Biostatistics, MRC-PHE Centre for Environment and Health, School of Public Health, Imperial College London, London, United Kingdom; 6 Cardiovascular Health Research Unit, Departments of Medicine, Epidemiology, and Health Services, University of Washington, Seattle, WA, United States of America; 7 Kaiser Permanente Washington Health Research Institute, Seattle, WA, United States of America; 8 Zilber School of Public Health, University of Wisconsin-Milwaukee, Milwaukee, WI, United States of America; 9 Department of Epidemiology, University of Washington, Seattle, WA, United States of America; 10 Division of Public Health Sciences, Fred Hutchinson Cancer Research Center, Seattle, WA, United States of America; Yale School of Medicine, UNITED STATES

## Abstract

The identification of rare coding or splice site variants remains the most straightforward strategy to link genes with human phenotypes. Here, we analyzed the association between 137,086 rare (minor allele frequency (MAF) <1%) coding or splice site variants and 15 hematological traits in up to 308,572 participants. We found 56 such rare coding or splice site variants at *P*<5x10^-8^, including 31 that are associated with a blood-cell phenotype for the first time. All but one of these 31 new independent variants map to loci previously implicated in hematopoiesis by genome-wide association studies (GWAS). This includes a rare splice acceptor variant (rs146597587, MAF = 0.5%) in interleukin 33 (*IL33*) associated with reduced eosinophil count (*P* = 2.4x10^-23^), and lower risk of asthma (*P* = 2.6x10^-7^, odds ratio [95% confidence interval] = 0.56 [0.45–0.70]) and allergic rhinitis (*P* = 4.2x10^-4^, odds ratio = 0.55 [0.39–0.76]). The single new locus identified in our study is defined by a rare p.Arg172Gly missense variant (rs145535174, MAF = 0.05%) in plasminogen (*PLG*) associated with increased platelet count (*P* = 6.8x10^-9^), and decreased D-dimer concentration (*P* = 0.018) and platelet reactivity (*P*<0.03). Finally, our results indicate that searching for rare coding or splice site variants in very large sample sizes can help prioritize causal genes at many GWAS loci associated with complex human diseases and traits.

## Author summary

Genome-wide association studies (GWAS) have identified thousand of genetic associations between common DNA sequence variants (*e*.*g*. single nucleotide polymorphisms (SNPs)) and complex human diseases or traits. In most cases, these associations highlight non-coding variants, and thus fall short of identifying causal genes. The discovery of rare coding variants within these GWAS loci can greatly simplify causal gene identification. Here, we tested the association between 137,086 rare coding and splice site variants (minor allele frequency (MAF) <1%) and 15 blood-cell traits in 308,572 participants. We found 56 rare coding variants associated with hematological traits, including 31 variants reported for the first time. Thirty of these 31 variants are located within blood-cell trait GWAS loci, thus prioritizing candidate genes. We replicated an association between a rare interleukin 33 (*IL33*) splice site variation and eosinophil count and asthma risk, and showed that it also associates with hay fever risk. Finally, we found a new rare missense variant in plasminogen (*PLG*) associated with platelet count, D-dimer concentration, and platelet reactivity. In conclusion, it is possible to use the “rare coding variant association study” strategy to pinpoint causal genes at GWAS loci, but very large sample sizes are required.

## Introduction

Although genome-wide association studies (GWAS) have identified thousands of associations with the number or distributional characteristics of red blood cells, white blood cells, and platelets [1], the genes responsible for this phenotypic variation remain elusive at most of these loci. For the most part, the difficulty in connecting association signals with genes resides in the fact that most GWAS variants are non-coding and in linkage disequilibrium (LD) with many other variants. This problem is not specific to hematological traits, but rather a general bottleneck in the functional characterization and clinical translation of most GWAS findings for common human diseases. Causal gene identification is important to shed light onto disease pathophysiology, but also to develop new, genetically-guided, therapeutic targets.

The discovery of rare variants that alter amino acid sequence or splicing is a strong indication that the mutated genes are involved in the phenotypes of interest. This approach is the cornerstone of Mendelian genetics. For complex human phenotypes, the discovery of rare coding variants associated with blood lipid levels [2, 3], coronary artery disease [4, 5], or more recently height [6] has helped prioritize causal genes and identify new drug targets (*e*.*g*. *PCSK9* for atherosclerosis). For blood-cell related phenotypes, the identification of rare missense variants in the chemokine receptor gene *CXCR2* [7] or the sphingosine-1 phosphate signalling gene *S1PR4* [8] has highlighted the importance that these biological pathways play in neutrophil count variation in humans.

Although extremely useful tools to pinpoint causal genes, the discovery of rare variants of modest effect sizes have previously been limited because they require very large sample sizes. Here, we took advantage of genotype data at 137,086 rare (minor allele frequency (MAF) <1%) variants in 308,572 participants to find new genes implicated in human hematopoiesis. We identified 56 rare coding variants, including 31 new variants, which highlight specific genes that contribute to inter-individual variation of red blood cell (RBC), white blood cells (WBC), and platelet (PLT) traits.

## Results

### 31 new rare coding or splice site variants associated with blood-cell traits

In an effort to identify rare coding (defined in this study as missense or nonsense, excluding synonymous) or splice site variants that could implicate new genes in human hematopoiesis, we performed meta-analyses at 137,086 variants for 15 blood-cell traits in 308,572 participants (**S1 Fig**). These analyses found 81 missense or splice site variants at *P*<5x10^-8^ with a minor allele frequency (MAF) <1% across the tested studies. We excluded 25 variants because they were not present in phase 1 of the Blood-Cell Consortium (BCX1) dataset (N = 2), the direction of effect was discordant across studies (N = 11), or they mapped to the HLA region (N = 15)(**S1 Table**). As positive controls to validate our approach, 25 rare coding or splice site variants previously associated with hematological traits were genome-wide significant in the current analyses (**S2 Table**) [1, 8–11]. In addition, we found 31 new rare coding or splice site variants in 29 different genes associated with blood-cell trait variation (**Table 1** and **S3 and S4 Tables**). The two variants in *ATR* are in strong linkage disequilibrium (LD), as are the two variants in *EXOC3L4* (D′>0.8).

**Table 1:** New rare coding or splice site variants associated with hematological traits. Chromosome and genomic coordinates are on build hg19 of the human genome. Allele frequencies and directions of effect are for allele A2. Effect sizes (BETA and SE) are in standard deviation units. *P*_het_ is the Cochran’s Q test heterogeneity P-value. We provide P-values in the UK Biobank (UKBB) without (non-conditional) and with (conditional) correction for the known blood-cell variants [1]. Detailed results of the conditional analyses are in **S6 Table**. Other phenotypes are hematological traits associated at *P*<5x10^-8^ in the meta-analyses. In the Comment column, we consider SNPs previously associated with blood-cell traits that are located 500 kb on either side of the reported rare variant. ^a^, variants that are statistically dependent of known blood-cell trait variants; ^b^, variants that are statistically independent from known blood-cell variants, but that are located at known blood-cell trait loci; ^c^, new blood-cell trait locus.

SNPID	CHR (POS)	Gene	Annotation (VEP)	A1/A2	Freq (A2)	Main phenotype	BETA (SE)	*P*-value	*P*_het_	UKBB *P*_non-cond_	UKBB *P*_cond_	Other phenotypes	Comment
rs148916169^a^	1 (36932463)	*CSF3R*	missense	A/G	0.0066	WBC	0.1308 (0.0181)	4.62E-13	0.16	6.09E-07	0.61	Neutro	Known WBC locus.
rs138903557^b^	2 (24245713)	*MFSD2B*	missense	C/G	0.0046	MCV	-0.126 (0.0231)	4.76E-08	0.48	2.70E-07	0.000032		Locus associated with WBC and RBC traits.
rs147820690^b^	2 (160735174)	*LY75-CD302*	missense	C/T	0.0026	PLT	0.1654 (0.0287)	8.19E-09	0.83	0.0043	0.00069		Locus associated with PLT and WBC traits.
rs116274727^b^	2 (192701265)	*SDPR*	missense	T/C	0.0061	MPV	0.1623 (0.0215)	3.95E-14	0.97	7.77E-08	9.57E-12		Known PLT locus.
rs28910273^b^	3 (142188337)	*ATR*	missense	A/C	0.0067	MCV	-0.1318 (0.0185)	1.02E-12	0.17	1.70E-08	3.74E-10	MCH	Known RBC locus.
rs77208665^b^	3 (142274770)	*ATR*	missense	T/C	0.0056	MCV	-0.1171 (0.0208)	1.91E-08	0.089	1.38E-05	5.16E-07		
rs151053159^b^	5 (1078832)	*SLC12A7*	missense	C/T	0.0034	RDW	-0.2013 (0.0278)	4.77E-13	0.99	9.81E-10	0.0012		Locus associated with WBC and RBC traits.
rs121434346^a^	5 (1212453)	*SLC6A19*	missense	G/A	0.0042	RDW	-0.1379 (0.0246)	2.15E-08	0.65	2.06E-05	0.63		Locus associated with WBC and RBC traits.
rs145535174^c^	6 (161134124)	*PLG*	missense	A/G	0.0005	PLT	0.34 (0.0587)	6.83E-09	1.0	0.00017	3.62E-05		Novel locus
rs74848966^a^	7 (100365467)	*ZAN*	missense	G/A	0.0044	MCH	0.1522 (0.0263)	7.02E-09	0.26	2.20E-06	0.27		*EPO*/*TFR2* locus, previously associated with RBC traits.
rs141547371^a^	9 (214606)	*C9orf66*	missense	A/C	0.0027	MPV	0.3022 (0.0479)	2.87E-10	0.37	5.12E-08	0.012		Known PLT locus.
rs146597587^a^	9 (6255967)	*IL33*	splice acceptor	G/C	0.0047	Eosin	-0.2504 (0.0252)	2.40E-23	0.12	1.02E-17	0.88		Known WBC locus.
rs146879704^b^	9 (114886569)	*SUSD1*	missense	C/T	0.0037	HGB	0.1509 (0.0254)	2.92E-09	0.16	1.87E-06	3.00E-05		Known RBC locus.
rs141547732^a^	9 (136280025)	*REXO4*	missense	G/A	0.0064	RBC	0.1195 (0.0195)	8.47E-10	0.59	8.05E-07	0.40		*ABO* locus, previously associated with RBC traits.
rs71508957^a^	10 (64927837)	*JMJD1C*	missense	C/T	0.0085	MPV	0.1142 (0.0175)	7.54E-11	0.19	4.14E-08	0.030	PLT	Locus associated with WBC, PLT and RBC traits.
rs61748606^a^	11 (230474)	*SIRT3*	missense	G/T	0.0060	MPV	-0.1624 (0.0274)	3.05E-09	0.32	3.74E-10	0.46		Locus associated with WBC, PLT and RBC traits.
rs138326449^b^	11 (116701354)	*APOC3*	splice donor	G/A	0.0020	RDW	0.2857 (0.0444)	1.19E-10	0.018	4.48E-08	2.72E-09		Known PLT locus. This rare variants is associated with triglyceride levels and coronary artery disease.
rs150349412^a^	12 (112184086)	*ACAD10*	missense	G/A	0.0027	PLT	0.176 (0.0318)	3.01E-08	0.32	2.19E-06	0.65		*SH2B3* locus, previously associated with RBC, WBC and PLT traits.
rs145120027^a^	12 (122439451)	*WDR66*	missense	T/C	0.0011	MPV	0.3647 (0.0591)	6.70E-10	0.039	2.02E-09	0.84		Locus associated with PLT and RBC traits.
rs17881033^a^	12 (122763670)	*CLIP1*	missense	C/A	0.0091	MPV	0.1792 (0.0171)	9.90E-26	0.087	2.44E-20	0.66		
rs151322438^a^	12 (123335398)	*HIP1R*	missense	C/T	0.0019	MPV	0.3258 (0.0407)	1.16E-15	0.26	1.21E-13	0.20		
rs146030737^a^	13 (28626716)	*FLT3*	missense	C/T	0.0057	Mono	0.1475 (0.0249)	3.15E-09	0.67	3.81E-06	0.52		Known WBC locus.
rs182782800^a^	13 (73319139)	*BORA*	missense	G/A	0.0038	MCV	-0.1802 (0.024)	5.50E-14	0.53	3.26E-09	0.012	MCH	Known RBC locus.
rs13888768^b^	14 (103568488)	*EXOC3L4*	missense	G/A	0.0023	MPV	-0.2783 (0.0468)	2.72E-09	0.23	6.37E-07	1.36E-10		Known PLT locus.
rs148718670^a^	14 (103574815)	*EXOC3L4*	missense	G/A	0.0032	MPV	-0.1677 (0.0295)	1.28E-08	0.057	4.24E-06	0.0034		
rs184575290^b^	15 (80191280)	*ST20*	missense	T/A	0.0035	Mono	0.2164 (0.0302)	7.28E-13	0.0048	1.57E-10	9.30E-09		Known WBC locus.
rs57268939^b^	16 (319547)	*FAM234A*	missense	T/C	0.0005	MCH	-0.5633 (0.0922)	9.83E-10	0.034	6.05E-11	1.85E-09	MCV	α-globin locus, previously associated with RBC traits.
rs147810715^b^	16 (30999491)	*HSD3B7*	missense	G/C	0.0004	MCH	-0.4918 (0.0838)	4.39E-09	0.63	3.22E-08	4.74E-08		Known RBC locus.
rs35266519 ^a^	17 (38062390)	*GSDMB*	missense	C/T	0.0089	Neutro	-0.101 (0.0173)	4.84E-09	0.61	3.18E-07	0.32		Known WBC locus.
rs150420714^b^	19 (50017538)	*FCGRT*	missense	G/C	0.0083	HCT	0.1033 (0.0167)	6.26E-10	0.43	9.77E-05	2.76E-05	HGB, RBC	Known RBC locus.
rs201148397^b^	22 (37482458)	*TMPRSS6*	missense	C/A	0.0021	MCH	0.2542 (0.0371)	7.12E-12	0.031	3.89E-09	8.86E-05	MCV	Locus associated with PLT and RBC traits.

All 31 variants are novel discoveries because they were not identified in previous large-scale association studies for blood-cell traits. However, although the variants are new, many of them map to known blood-cell trait loci. Indeed, using conditional analyses in the UK Biobank, we could sub-divide these 31 variants into three categories: 16 variants are in LD with markers previously reported to associate with hematological traits (*P*_cond_ >0.002 in **Table 1** and **S5 and S6 Tables**), 14 variants are not in LD with known blood-cell variants, but are located within 500 kb of one of these previously reported variants, and one variant (*PLG*-rs145535174) defines a new locus associated with PLT count variation in humans.

### A rare missense variant in plasminogen associates with platelet count, D-dimer concentration, and platelet reactivity

Plasminogen (*PLG*) encodes the precursor of plasmin, an important proteolytic enzyme that degrades many plasma proteins and fibrin blood clots. The rare G-allele at this new *PLG* variant (rs145535174, MAF = 0.05%) is associated with increased PLT count (+20x10^9^ platelets/l, *P* = 6.8x10^-9^). It replaces an arginine residue at position 172 into a glycine, an amino acid change that is predicted to be probably damaging by Polyphen and deleterious by SIFT [12, 13]. This genomic position is highly conserved, as evidenced by a GERP score >200 and a CADD score of 23.8, ranking this particular amino acid change in *PLG* among the top 1% of the most deleterious substitutions in the human genome [14].

Given the role of plasmin in hemostasis, we tested the association between rs145535174 and several hemostatic and coagulation factors in the FHS. Although we only found a maximum of 16 carriers of the rare allele (varying depending on overlapping phenotypes), we could detect nominal associations between rs145535174-G and decreased D-dimer concentration and platelet reactivity (**Table 2**). Because D-dimer is a by-product of fibrin clot degradation by plasmin, this result is consistent with rs145535174-G being a *PLG* loss-of-function variant. Since plasminogen/plasmin activity also plays a role in thrombotic diseases, we tested if the G-allele at *PLG*-rs145535174 increases the risk of myocardial infarction (MI), stroke, or venous thromboembolism (VTE) in the UK Biobank (**S7 Table**). This variant was not associated with MI or stroke risk (*P*>0.05). However, there was an association with increased VTE risk in the UK Biobank (*P* = 0.0087, odds ratio = 4.01), although it was not replicated in the Montreal Heart Institute Biobank and the Women’s Health Initiative (*P*>0.05, **S7 Table**). The absence of genetic associations between *PLG*-rs145535174 and thrombotic events could simply reflect our limited statistical power given the rarity of the G-allele.

**Table 2:** Association results between *PLG*-rs145535174 and hemostatic and coagulation factors in the Framingham Heart Study. The direction of the effects (BETA) is given for the rare G-allele (on forward strand). SE, standard error; ADP, Adenosine diphosphate; PAI-1, plasminogen activator inhibitor type 1; tPA, tissue plasminogen activator; FVII, factor VII; vWF, von Willebrand factor. We highlight in bold nominal P-values <0.05.

Trait	Sample size (# carriers)	Freq (G-allele)	BETA (SE)	*P*-value
Epinephrine 0.5uM Platelet Reactivity, % aggregation	1877 (6)	0.0016	-1.092 (0.432)	**0.012**
Epinephrine 1.0uM Platelet Reactivity, % aggregation	2009 (7)	0.0017	-0.774 (0.402)	0.054
Epinephrine 3.0uM Platelet Reactivity, % aggregation	1131 (6)	0.0027	-0.286(0.426)	0.502
Epinephrine EC50 Platelet Reactivity, *u*M	2199 (8)	0.0018	0.737 (0.381)	0.053
Collagen lag time, second	2152 (8)	0.0019	0.435 (0.383)	0.256
ADP 1.0uM Platelet Reactivity, % aggregation	2392 (7)	0.0015	-0.920 (0.415)	**0.026**
ADP 3.0uM Platelet Reactivity, % aggregation	2555 (8)	0.0016	-0.451 (0.375)	0.229
ADP 5.0uM Platelet Reactivity, % aggregation	1686 (7)	0.0021	-0.426 (0.408)	0.297
ADP EC50 Platelet Reactivity, *u*M	2205 (8)	0.0018	0.3818 (0.378)	0.313
PAI-1, ng/ml	6441 (14)	0.0011	-0.339 (0.197)	0.086
tPA, ng/ml	2613 (9)	0.0017	-0.116 (0.152)	0.446
D-dimer, ng/ml	2874 (8)	0.0005	-0.479 (0.202)	**0.018**
Fibrinogen, g/L	6711 (16)	0.0012	-0.059 (0.047)	0.207
FVII, % antigen	2620 (9)	0.0017	2.261 (5.563)	0.684
vWF % antigen	2621 (9)	0.0017	21.187 (14.551)	0.145

### Blood-cell trait-associated rare variants prioritize genes at GWAS loci

When we considered genes with known hematopoietic functions, we found little evidence that the rare coding or splice site variants found here implicated different potentially causal genes than candidate genes from previous blood-cell trait GWAS (**S8 Table**). In other words, we have, with one exception, no loci with two strong blood-cell trait candidate genes. On chromosome 11, we found an association between mean platelet volume (MPV) and a rare coding variant in *SIRT3*, a gene implicated in platelet biology [15]. At the same locus, Gieger et *al*. had nominated *PSMD13* as a candidate platelet gene on the basis of reduction in plasmatocyte numbers in *Drosophila* [16]. We also noted few examples where rare coding or splice site variants implicated the same genes than those prioritized by GWAS SNPs due to expression quantitative trait loci effects (*e*.*g*. *SLC12A7*, *FLT3*, *TMPRSS6*)(**S8 Table**).

Conditional analyses identified 16 rare variants in LD with previously reported blood-cell variants (**S5 and S6 Tables**). Despite this dependence due to LD, at some of these loci, we may have identified a rare variant with even greater biologic plausibility for the blood-cell trait association than the previously identified LD surrogate. Indeed, whereas all 16 variants from our study are coding or splice site variants, most LD surrogate variants are non-coding variants (**S6 Table**). To clarify whether the new (**Table 1**) or previously known [1] blood-cell trait variants are more likely causal variants, we performed the reciprocal conditional analyses in which we conditioned the previously known blood-cell trait variants on genotypes at the newly described 31 rare coding or splice site variants (**S9 Table**). Comparison of the conditional results (**S6** and **S9 Tables**) highlighted a few loci where the common variants are better causal candidates (*e*.*g*. *WDR66*, *CLIP1*, *HIP1R*). There were also a few loci where the new rare coding or splice site variants were statistically equivalent to rare non-coding variants previously reported (*e*.*g*. *IL33*, *FLT3*, *BORA*). We did not find examples of rare variants completely explaining the association signals at known common variants, as we would expect given limited LD.

On chromosome 1, we found a rare missense variant in *CSF3R* (rs148916169, MAF = 0.7%) associated with total WBC and neutrophil counts. Although this variant is correlated with common and low-frequency intronic variants (**S6 Table**), it is the first missense variant to directly implicate *CSF3R* in WBC count variation in the general population. *CSF3R* encodes the receptor for CSF3, a cytokine that controls the proliferation and differentiation of granulocytes, and is also mutated in a severe form of congenital neutropenia (MIM# 617014) [17]. Although rare loss-of-function mutations in *CSF3R* are associated with reduced neutrophil levels, the rare *CSF3R* missense variant found in our experiment might represent a gain-of-function since its rare allele is associated with higher WBC and neutrophil counts.

Another compelling example is the identification of a rare splice acceptor variant in *IL33* (rs146597587, MAF = 0.5%) associated with reduced eosinophil count (**Table 1**). This likely functional *IL33* variant maps to a locus with three non-coding variants, including a rare intergenic variant, associated with WBC traits (**S6 Table**) [1]. Interestingly, GWAS have associated *IL33* common non-coding variants with risk for asthma [18], allergic rhinitis [19], and endometriosis [20]. In the UK Biobank, we found that the rare C-allele at *IL33*-rs146597587 associated with reduced eosinophil count is also associated with lower risk of asthma (*P* = 2.60x10^-7^, odds ratio = 0.56) and allergic rhinitis (*P* = 4.21x10^-4^, odds ratio = 0.55) (**Table 3**). These associations remained significant after accounting for the known GWAS variants previously identified at this locus (**Table 3**). Our finding strongly reinforces the clinical importance that eosinophils play in the pathophysiology of these diseases, and highlight the *IL33* pathway as a promising therapeutic target. However, in a mediation analysis in the UK Biobank in which we corrected for eosinophil count, we found that the protective effect of *IL33*-rs146597587 was weaker but remained significant (*P*_mediation_ = 3.4x10^-5^). This suggests that *IL33* may influence asthma risk in part by controlling blood eosinophil count, but also through currently unknown additional biological pathways. Finally, we note that the same rare *IL33* variant was recently shown to associate with reduced eosinophil count and protection from asthma in the Icelandic population [21]. Because there is no sample overlap, our study represents a strong replication of this original report: in a meta-analysis of both studies, association results now reach genome-wide significance (in 24,030 asthma cases and 421,096 controls, *P* = 1.4x10^-10^, odds ratio = 0.54).

**Table 3:** Association between *IL33*-rs146597587 and asthma, allergic rhinitis, or endometriosis risk in the UK Biobank. All analyses were corrected for age, sex, and the first 10 principal components. The direction of the odds ratio is for the rare allele (rs146597587, C-allele on forward strand). The conditional *P*-values were calculated in a logistic model accounting for genotypes at common single nucleotide polymorphisms (SNPs) previously associated with asthma, allergic rhinitis, or endometriosis: rs343496, rs7032572, rs72699186, rs1342326, rs2381416, rs928413, rs10975519.

Disease	N_cases_	N_ctrls_	Freq (C-allele)	Odds ratio	95% confidence interval	*P*-value	*P*-value (conditional)
Asthma	17,565	118,119	0.0048	0.56	0.45–0.70	2.60x10^-7^	1.22x10^-6^
Allergic rhinitis	7,918	127,766	0.0048	0.55	0.39–0.76	4.21x10^-4^	7.4x10^-4^
Endometriosis	1,589	134,095	0.0048	0.86	0.46–1.49	0.63	0.67

We found 14 rare coding or splice site variants that appear to be associated with blood-cell traits independently of other common and/or rare variants previously identified (**Table 1** and **S6 Table**). Although there are a few well-known blood-cell-related genes, such as *SLC12A7* and *TMPRSS6*, most of the highlighted genes do not have assigned functions in hematopoiesis (*e*.*g*. *MFSD2B*, *SDPR*, C*9orf66*, *SUSD1*, *EXOC3L4*)(**Table 1**). We found two correlated rare missense variants in *ATR* (rs28910273, MAF = 0.6%; rs77208665, MAF = 0.7%), which encodes a checkpoint protein that coordinates cellular responses to replication stress and DNA damage. *ATR* is mutated in Seckel syndrome 1 (MIM #210600), which is characterized by hematological defects in some patients [22]. We also uncovered a rare splice acceptor variant in the apolipoprotein gene *APOC3* (rs138326449, MAF = 0.2%) associated with RDW, a RBC trait that is considered a non-specific inflammatory marker and a predictor of cardiovascular diseases [23]. The same *APOC3* variant has previously been associated with lower triglyceride levels and coronary artery disease risk [5, 24].

### Biological pathway analyses with blood-cell trait genes

We performed three biological pathway-enrichment analyses using 58 genes that harbour a rare coding or splice site variants associated with RBC, WBC, or PLT traits (**Table 1** and **S2 Table**). In total, we found six, 13, and five biological pathways or terms enriched for genes with variants associated with RBC, WBC, and PLT, respectively, at a false discovery rate <10% (**Table 4**). Most of these pathways were highly redundant in terms of gene content. For instance, five of the six RBC-enriched pathways are involved in iron homeostasis. For WBC traits, there were many significant inter-connected biological pathways that implicated six genes: *CSF3R*, *CXCR2*, *S1PR4*, *IL17RA*, *AMICA1/JAML*, and *FLT3* (**Table 4**). These genes highlight the importance that genetic variation plays on cytokine signalling and chemotaxis to modulate circulating numbers of immune cells in the blood.

**Table 4:** Pathway analyses using DAVID with genes that carry rare coding or splice site variants associated with blood-cell traits. We only present biological pathways with a false discovery rate (FDR) <10%.

Biological pathway or term	Fold Enrichment	P-value	FDR	Genes
***Red blood cell***				
Iron ion homeostasis	121.3	0.00023	0.26	*TF*, *TFR2*, *TMPRSS6*
Cellular iron ion homeostasis	85.3	0.00046	0.53	*TF*, *TFR2*, *TMPRSS6*
Ferrous iron import into cell	1051.5	0.00175	1.99	*TF*, *TFR2*
Cellular response to iron ion	525.8	0.00351	3.95	*TF*, *TFR2*
HFE-transferrin receptor complex	279.3	0.00667	6.00	*TF*, *TFR2*
Disulfide bond	3.2	0.00895	9.32	*TF*, *TFR2*, *SUSD1*, *ZAN*, *FCGRT*, *TMPRSS6*, *EPO*
***White blood cells***				
Cytokine-cytokine receptor interaction	19.1	0.00040	0.28	*FLT3*, *CSF3R*, *CXCR2*, *IL17RA*
Neutrophil chemotaxis	99.1	0.00031	0.34	*CSF3R*, *CXCR2*, *JAML*
Cytokine-mediated signaling pathway	41.1	0.00177	1.94	*FLT3*, *CSF3R*, *IL17RA*
Integral component of plasma membrane	6.6	0.00272	1.98	*FLT3*, *S1PR4*, *CSF3R*, *CXCR2*, *IL17RA*
Plasma membrane	3.3	0.00294	2.15	*GSDMB*, *FLT3*, *S1PR4*, *CSF3R*, *CXCR2*, *JAML*, *IL17RA*
Receptor	6.4	0.00292	2.63	*FLT3*, *S1PR4*, *CSF3R*, *CXCR2*, *IL17RA*
Topological domain:Extracellular	4.3	0.00316	2.72	*FLT3*, *S1PR4*, *CSF3R*, *CXCR2*, *JAML*, *IL17RA*
Cell membrane	4.0	0.00461	4.11	*GSDMB*, *S1PR4*, *CSF3R*, *CXCR2*, *JAML*, *IL17RA*
Disulfide bond	3.7	0.00632	5.60	*GSDMB*, *FLT3*, *CSF3R*, *CXCR2*, *JAML*, *IL17RA*
Membrane	2.3	0.00732	6.46	*GSDMB*, *FLT3*, *S1PR4*, *CSF3R*, *CXCR2*, *JAML*, *IL33*, *IL17RA*
Topological domain:Cytoplasmic	3.5	0.00833	7.04	*FLT3*, *S1PR4*, *CSF3R*, *CXCR2*, *JAML*, *IL17RA*
Transmembrane helix	2.6	0.01030	8.99	*FLT3*, *S1PR4*, *CSF3R*, *CXCR2*, *JAML*, *IL33*, *IL17RA*
Transmembrane	2.6	0.01043	9.10	*FLT3*, *S1PR4*, *CSF3R*, *CXCR2*, *JAML*, *IL33*, *IL17RA*
***Platelet***				
Blood coagulation	26.4	0.00033	0.40	*SH2B3*, *JAK2*, *JMJD1C*, *PLG*
Erythrocytosis, somatic	505.5	0.00329	2.10	*SH2B3*, *JAK2*
Myelofibrosis, somatic	337.0	0.00494	3.14	*SH2B3*, *JAK2*
Pleckstrin homology-like domain	9.4	0.00682	7.13	*PLEK*, *SH2B3*, *JAK2*, *KALRN*
Phosphoprotein	1.8	0.00723	7.31	*PLEK*, *HIP1R*, *IQGAP2*, *PLG*, *EXOC3L4*, *CKAP2L*, *SDPR*, *CLIP1*, *SH2B3*, *JAK2*, *JMJD1C*, *TUBB1*, *LY75-CD302*, *KALRN*

## Discussion

Using a large sample of >300,000 participants, we identified 31 new missense or splice site variants associated with blood-cell traits. These include a rare p.Arg172Gly missense of plasminogen associated with higher platelet count, lower D-dimer concentration and lower platelet reactivity and a rare splice acceptor variant of *IL33* associated with lower eosinophil count and lower risk of asthma and allergic rhinitis. At other genomic loci, our findings may prioritize the causal gene(s) that can be pursued with molecular techniques to further dissect and define the mechanism of association. Because phenotypic variance explained is determined by the effect size and allele frequency of the variants, the rare coding and splice site variants identified here do not have a large contribution to the heritability of blood-cell traits. For instance, the rare *IL33*-rs146597587 variant explains only 0.06% of the variation in eosinophil count.

The chromosome 2p23 region spans several genes and contains six common, non-coding variants associated previously with WBC or RBC traits [1]. We identified a new rare coding variant in this region associated with lower MCV located in *MFSD2B*, a transporter gene of unknown function that is highly and specifically expressed in blood and bone marrow cells, particularly of the erythroid lineage [25]. As another example, at the *LY75-CD302* locus, four common non-coding variants and a low-frequency missense variant have been associated with PLT and WBC traits [10]. A second novel rare *LY75-CD302* missense variant was associated with higher PLT count. The *LY75-CD302* locus represents a naturally occurring read-through transcript between the lymphocyte antigen 75 (*LY75*) and *CD302* genes, and is expressed in leukocyte and hematopoietic stem and progenitor cells [25]. Alternative splicing results in fusion *LY75*-*CD302* gene products that are expressed during dendritic cell maturation [26] and Hodgkin’s lymphoma cell lines [27]. Another example of a potential read-through transcript involves a gene-rich region on chromosome 15, where several common variants were recently associated with WBC traits [1]. At this locus, we discovered a rare coding variant (rs184575290) located near the *ST20* termination codon associated with higher monocyte count. This locus represents a naturally occurring read-through transcript that produces a fusion protein between *ST20* and the neighboring *MTHFS* gene. *ST20* is highly expressed in myeloid cells, while the fusion product is expressed at lower levels in blood neutrophils and dendritic cells of the skin [25].

We found a rare missense variant within *SDPR*, a phosphatidylserine-binding protein originally isolated from human platelets [28], that may be involved in modulating activation of the platelet protein kinase C substrate pleckstrin and mediating the downstream effects of platelet granule secretion [29]. Common missense variants of *EXOC3L4*, which has no known function, have been previously associated with both platelet and liver enzyme traits [30]. In progenitor blood-cell expression data from the BLUEPRINT Project [31], there is a large increase in *EXOC3L4* expression in megakaryocyte progenitors. Several rare, non-coding variants at the chromosome 13q21 region have been associated with RBC traits [1]. We identified a rare coding variant of *BORA*, an activator of the protein kinase Aurora A involved in centrosome maturation and spindle assembly during mitosis and whose expression pattern during hematopoiesis increases in RBC and PLT precursors [31].

Matriptase-2, encoded by *TMPRSS6*, has recently emerged through both complex trait and Mendelian disorders as an important genetic regulator of RBC and iron metabolism [32]. We report here the first rare coding variant of *TMPRSS6* (p.V280L/p.V289L) associated with RBC traits in individuals unselected for hematologic disease. Other *TMPRSS6* coding variants have been reported in patients with familial iron-refractory iron-deficiency anemia (IRIDA), a rare autosomal-recessive disorder characterized by hypochromic microcytic anemia and impaired iron balance [33].

Plasminogen plays an important role in fibrinolysis as well as wound healing, cell migration, and tissue re-modeling. Congenital plasminogen deficiency is a rare autosomal recessive disorder [34]. Severely affected individuals (type I plasminogen deficiency) can exhibit defective extracellular fibrin clearance during wound healing, leading to “ligneous conjunctivitis'' or thick, pseudomembranes on conjunctival and other mucosal surfaces. The p.Arg172Gly variant of plasminogen associated with higher PLT count is located in the first kringle domain, which mediates binding of plasminogen to fibrin and cell surfaces [35]. The observed association of the *PLG* p.Arg172Gly variant with lower D-dimer is consistent with reduced fibrinolysis, which might result from reduced circulating plasminogen, plasmin activity, or substrate binding. Overall, we did not observe an association between *PLG* p.Arg172Gly and risk of thrombotic disease. Similarly, mutations associated with congenital plasminogen deficiency do not appear to be a strong risk factor for VTE [36–38].

The reasons for the association of the *PLG* p.Arg172Gly variant with higher PLT count and lower platelet reactivity are not readily apparent. *In vitro*, plasmin has been reported to proteolytically inactivate thrombopoietin [39]. Plasmin also is capable of activating platelets through protease-activated receptors (PAR)-1 and -4 [40, 41]. Thus, it is possible that the *PLG* loss-of-function variant may influence thrombopoietin-induced platelet production, or PAR-induced platelet aggregation, respectively. Finally, it is worth noting that moderately reduced platelet count is a feature of a congenital platelet disorder characterized by a gain-of-function fibrinolysis defect due to increased expression and storage of urokinase plasminogen activator (*PLAU*) during megakaryocyte differentiation [42].

In conclusion, focusing on 137,086 rare coding or splice site variants in 308,572 participants, we discovered 31 new variants associated with blood-cell traits. Thirty of these 31 variants map to loci previously implicated by GWAS for hematological phenotypes. Because we used the ExomeChip or the UK Biobank array, there is an enrichment of GWAS signals among the loci tested, although many of the loci identified here did not harbour blood-cell trait genetic associations at the time the arrays were designed. As discussed, many of these associations prioritize strong candidate genes at these loci. Our study was limited by the content of the genotyping arrays utilized. As we expand our genetic experiments to complete genome sequence in very large cohorts, we anticipate that we will uncover more rare coding variants and, maybe more importantly for gene identification within GWAS loci, additional independent variants in the same candidate genes. Together with a recent report for adult height [6], our findings reinforce the importance that rare variants play in the architecture of complex human phenotypes.

## Methods

### Ethical statement

Written, informed consent was obtained for all participants in accordance with the Declaration of Helsinki. This project was also reviewed and approved by the Montreal Heart Institute Ethics Committee and the different recruiting centers (approval number 2014–1707).

### Statistical analyses

Single variant association results considered in this effort were obtained from participants of European ancestry using an additive genetic model. All phenotypes were corrected for confounding factors (see below) and normalized using inverse normal transformation. The final analyses included samples from BCX1 (up to 157,622 participants), AIRWAVE (up to 14,866), and the first release of the UK Biobank (up to 136,084). Genotypes for BCX1 and AIRWAVE were obtained from the Illumina ExomeChip array; the content is available at: http://genome.sph.umich.edu/wiki/Exome_Chip_Design. The ExomeChip includes ~250,000 exonic variants obtained from whole-exome sequencing of ~12,000 participants. The UK Biobank samples were genotyped on the UK Biobank Axiom array; the content of the arrays is available at: http://www.ukbiobank.ac.uk/scientists-3/uk-biobank-axiom-array/. Whereas the Axiom array targets >800,000 variants, we only analyzed exonic variants that overlap with the content of the ExomeChip.

Phenotypic information, and ExomeChip quality-control steps and association results from cohorts involved in the BCX1 Consortium and AIRWAVE have been described elsewhere [10, 11, 43]. Briefly, we excluded variants with low genotyping success rate (<95%, except for WHI that used a cutoff <90%). Samples with call rate <95% after joint or zCALL calling and with outlying heterozygosity rate were also excluded. Other exclusions were deviation from Hardy-Weinberg equilibrium (*P*<1x10^-6^) and gender mismatch. We performed principal component analysis (PCA) or multidimensional scaling (MDS) and excluded sample outliers from the resulting plots, using populations from the 1000 Genomes Project to anchor these analyses. Prior to performing meta-analyses of the association results provided by each participating study, we ran the EasyQC protocol [44] to check for population allele frequency deviations and proper trait transformation in each cohort. In terms of hematological phenotypes, we excluded individuals with blood cancer, leukemia, lymphoma, bone marrow transplant, congenital or hereditary anemia, HIV, end-stage kidney disease, dialysis, splenectomy, and cirrhosis, and those with extreme measurements of RBC traits. We also excluded individuals on erythropoietin treatment or chemotherapy. Additionally, we excluded pregnant women and individuals with acute medical illness at the time the complete blood count (CBC) was done. For all traits, within each study, we adjusted for age, age-squared, gender, the first 10 principal components, and, where applicable, other study-specific covariates such as study center using a linear regression model. Within each study, we then applied inverse normal transformation on the residuals and tested the phenotypes for association with the ExomeChip variants using either RVtests (version 20140416) [45] or RAREMETALWORKER.0.4.9 [46].

A description of methods and quality-control procedures for the blood-cell data for the first release of the UK Biobank can also be found elsewhere [1]. Description of the exome-component of the genotyping arrays used for the UK Biobank samples can be found at: http://www.ukbiobank.ac.uk/scientists-3/uk-biobank-axiom-array/. In the UK Biobank, we modelled blood-cell traits using the following covariates: age, sex, menopause status for women, assessment centre where the blood samples were collected, machine counter that processed the blood samples, month, day of the week, time inside the day that the samples were collected, self-reported ethnic background of the individuals, smoking status and smoking frequency, alcohol drinker status, alcohol intake frequency, height and weight. In the first release of the UK Biobank, we tested the association between directly genotyped or imputed variants and normalized hematological traits with BOLT-LMM [47].

We meta-analyzed results from BCX1, AIRWAVE and the UK Biobank using inverse-variance weighting as implemented in METAL [48]. We limited our analyses to variants with a mean minor allele frequency (MAF) <1% in the meta-analyses that are annotated as coding (missense or nonsense) or splice site (acceptor or donor) using ENSEMBL’s Variant Effect Predictor (VEP). Furthermore, the variants had to be present on the Illumina exome array used by the BCX1 studies. In total, we tested 137,086 variants against 15 blood-cell traits. These phenotypes are divided between the main three cell types found in blood: red blood cells (red blood cell count (RBC count), hemoglobin concentration (HGB), hematocrit (HCT), mean corpuscular hemoglobin (MCH), mean corpuscular volume (MCV), mean corpuscular hemoglobin concentration (MCHC), and RBC distribution width (RDW)), white blood cells (total white blood cell count (WBC count), neutrophil count (Neutro), lymphocyte count (Lympho), monocyte count (Mono), basophil count (Baso), and eosinophil count (Eosin)), and platelets (platelet count (PLT count) and mean platelet volume (MPV)). The meta-analysis results are available at: http://www.mhi-humangenetics.org/en/resources. We used a conservative α = 5x10^-8^ to declare statistical significance. At this statistical threshold, our sample size (N = 308,572) provides >99% power to discover variants with MAF = 0.1% that explain >0.03% of the phenotypic variance.

### Conditional analyses

To test whether the 31 rare variants newly identified in the meta-analyses are associated with blood-cell traits independently of other known genetic loci, we regressed out the effect of the known blood-cell variants previously identified in the first release of the UK Biobank [1] from the normalized blood-cell phenotypes. All these analyses were done per phenotype; that is we fitted 15 different models for each of the 15 blood-cell phenotypes tested. We then re-tested in the UK Biobank (using linear regression implemented in R) the association between the “residual” blood-cell phenotypes and genotypes at the rare variants identified in the meta-analyses. For instance, for hemoglobin, we conditioned the new rare coding or splice site hemoglobin variants on all variants (across the genome) previously reported to associate with hemoglobin levels. We provide the complete list of variants on which we conditioned in **S5 Table** per blood-cell trait. If the rare variants were not significantly associated with the residual phenotypes, we then ran pairwise conditional analyses to identify which previously known variants at the locus accounted for the association signal identified in this project. To declare statistical independence from previously reported hematological trait association signals, we used α = 0.002 (Bonferroni correction for 31 variants tested).

### Secondary analyses for the rare PLG-rs145535174 variant

We sought association of the *PLG* variant (rs145535174) with platelet reactivity, as well as hemostatic and coagulation factors in the Framingham Heart Study (FHS) [49]. Genotyping for rs145535174 was conducted using the Illumina Human Exome BeadChip v.1.0 (Illumina, Inc., San Diego, CA) [50]. Multiple measurements were assessed for platelet reactivity [51], including maximum percentage platelet aggregation in response to agonists, *i*.*e*. ADP and epinephrine; minimal concentration of each agonist to produce a >50% aggregation response (EC50); and lag time in response to collagen stimulation. Hemostatic factors and coagulation factors, including antigens of plasminogen activator inhibitor-1 (PAI-1), tissue plasminogen activator (tPA), D-dimer, clotting factor VII (FVII) and von Willebrand factor (VWF), were measured using enzyme-linked immunosorbent assays [52–54] while fibrinogen levels were assessed using the Clauss method [52]. Association analyses in the FHS were conducted using either RAREMETALWORKER (http://genome.sph.umich.edu/wiki/RAREMETALWORKER) or seqMeta (http://cran.r-project.org/web/packages/seqMeta/index.html). A linear mixed effects model that accounts for familial correlation was used and adjustments were made for age, sex and principal components. The phenotypes were log-transformed or inverse normal transformed, as needed.

We tested the association between the rare G-allele at *PLG*-rs145535174 and myocardial infarction (MI) and stroke in the UK Biobank. We used the “Health and Medical History” records to identify MI and stroke cases. For MI, we used the search terms “heart attack”, “myocardial infarction”, “acute myocardial infarction”, “subsequent myocardial infarction” and “old myocardial infarction” to retrieve affected individuals. We used the terms “stroke”, “ischaemic stroke” and “cerebral infarction” to define stroke cases. As controls, we excluded UK Biobank participants with MI, stroke or transient ischemic attack, percutaneous coronary intervention, coronary artery bypass graft surgery, peripheral vascular disease, congestive heart failure, and angina. For analysis of venous thromboembolism (VTE), we identified cases in the UK Biobank, the Montreal Heart Institute Biobank, and the Women’s Health Initiative as individuals with pulmonary embolism or deep vein thrombosis. We tested the genetic association by logistic regression in PLINK or R, correcting for age, sex and the first ten principal components when available.

### Secondary analyses for the rare IL33-rs146597587 variant

We identified asthma, allergic rhinitis (hay fever), and endometriosis cases using the detailed “Health and Medical History” UK Biobank participant records. All other individuals were assigned as controls. We tested the genetic association by logistic regression in R, correcting for age, sex and the first ten principal components. To determine if the rare *IL33*-rs146597587 variant is independent from the previously reported common SNPs at the locus, we conditioned on genotypes at these variants (rs343496, rs7032572, rs72699186, rs1342326, rs2381416, rs928413, rs10975519) and re-run the logistic regression model.

### Pathway analyses

We used the default parameters in DAVID [55] to perform biological term and pathway enrichment analyses. For these bioinformatic analyses, we used as reference set all genes with at least one rare coding or splice site variant tested in the meta-analyses. We retrieved genes associated with RBC, WBC, or PLT traits from **Table 1** (new independent variants from our study) and **S2 Table** (known positive controls), and tested their enrichment in biological pathways in comparison with the reference set. Due to the relatively low number of genes that were used as input for this kind of analysis, we lowered the initial, minimum number of genes in a seeding group to 3 (default = 4) to ensure that the clustering algorithm will include as many genes as possible into functional groups. All other parameters were left at their default values.

## Supporting information

S1 Text Blood-Cell Consortium (BCX) membership and additional funding information. Additional funding information for the following studies: MHI Biobank, BioMe, Health 2006/2008, GeneSTAR, BIOVU, MESA, ARIC, JHS, WHI, HANDLS, GSK-STABILITY, SOLID TIMI-52, CHS, Framingham Heart Study, SHIP/SHIP-TREND, NWIGM, FINCAVAS, YFS, CARDIA, HABC, EGCUT, AIRWAVE.
(DOCX)

S1 Table Coding or splice site variants with a minor allele frequency <1% that were excluded during quality-control. Chromosomes and positions are on build hg19 of the human genome. The direction of the effect sizes (Beta) is for allele A2. Beta and standard errors (SE) are in standard deviation units. Mono, monocyte; WBC, white blood cell count; MCH, mean corpuscular hemoglobin; Lympho, lymphocyte; MPV, mean platelet volume; HGB, hemoglobin; RBC, red blood cell count; Baso, basophil.
(DOCX)

S2 Table Coding or splice site variants with a minor allele frequency <1% that were previously reported to associate with blood-cell traits. Chromosomes and positions are on build hg19 of the human genome. The direction of the effect sizes (Beta) is for allele A2. Beta and standard errors (SE) are in standard deviation units. MPV, mean platelet volume; MCH, mean corpuscular hemoglobin; RDW, red blood cell distribution width; PLT, platelet count; WBC, white blood cell count; MCHC, mean corpuscular hemoglobin concentration; Mono, monocyte; Neutro, neutrophil; HGB, hemoglobin.
(DOCX)

S3 Table Study-level association results for the 31 novel rare or splice site variants. The direction of the BETA is for the A2 allele. There are slight differences between the UK Biobank association results presented here and those in **Table 1** because of the model used for conditional analyses in **Table 1**.
(XLSX)

S4 Table Functional annotation of the 31 new independent rare (minor allele frequency <1%) variants identified in this study that are associated with hematological traits. (XLSX)

S5 Table List of variants for condtional analyses. For each blood-cell trait, we conditioned the association of the novel rare or coding variants on genotypes at these known blood-cell trait variants.
(XLSX)

S6 Table Conditional results in the UK Biobank (UKBB). When *P*_cond_ >0.002 (Bonferroni correction for 31 variants tested), we performed pairwise conditional analyses with markers at the locus to identify the variant that account for the association signal. This variant is listed in the Tagging variant column, along with its minor allele frequency and functional annotation. MPV, mean platelet volume; MCH, mean corpuscular hemoglobin; RDW, red blood cell distribution width; PLT, platelet count; WBC, white blood cell count; MCHC, mean corpuscular hemoglobin concentration; Mono, monocyte; Neutro, neutrophil; HGB, hemoglobin; MCV, mean corpuscular volume; Eosin, eosinophil; RBC, red blood cell count; HCT, hematocrit.
(DOCX)

S7 Table Association between *PLG*-rs145535174 and thrombotic events. All analyses were corrected for age and sex. UKBB, UK Biobank; MHIBB, Montreal Heart Institute Biobank; WHI, Women’s Health Initiative. The direction of the odds ratios is for the rare G-allele at rs145535174 (allele A2).
(DOCX)

S8 Table Prioritization of candidate genes. For each of the 31 novel rare coding or splice site variants presented in this study, we queried the corresponding loci in previous GWAS of blood-cell traits and highlighted previously prioritized candidate genes based on functional annotation (missense, splice site) or regulatory (eQTL) effect in the GTEx database.
(DOCX)

S9 Table Reciprocal conditional analysis results in the UK Biobank (UKBB). For each of the 31 variants in Table 1, we conditioned the variants from Astle et al. Associated with the same trait and located on the same chromosome. For instance for the IL33 locus, we conditioned all eosinophil-associated SNPs on chromosome 9 that were reported by Astle et al. with genotypes at the rare IL33 splice site variant (rs146597587).
(XLSX)

S1 Fig Quantile-quantile plots for rare-variant association results (minor allele frequency <1%). We present association results for 15 hematological traits analyzed at 137,086 variants in up to 308,572 participants.
(JPG)
