# Supplementary material for: Rare coding variants pinpoint genes that control human hematological traits
Source: PLoS Genet. 2017 Aug 7;13(8):e1006925. doi: 10.1371/journal.pgen.1006925 (PMC5560754; doi:10.1371/journal.pgen.1006925)
Supplement: S1 Text — Additional funding information for the following studies: MHI Biobank, BioMe, Health 2006/2008, GeneSTAR, BIOVU, MESA, ARIC, JHS, WHI, HANDLS, GSK-STABILITY, SOLID TIMI-52, CHS, Framingham Heart Study, SHIP/SHIP-TREND, NWIGM, FINCAVAS, YFS, CARDIA, HABC, EGCUT, AIRWAVE. (DOCX) [file pgen.1006925.s001.docx]

**SUPPLEMENTARY NOTE**

**Rare coding variants pinpoint genes that control human hematological traits**

Abdou Mousas, Georgios Ntritsos, Ming-Huei Chen, Ci Song, Jennifer E. Huffman, Ioanna Tzoulaki, Paul Elliott, Bruce M. Psaty, Blood-Cell Consortium, Paul L. Auer, Andrew D. Johnson, Evangelos Evangelou, Guillaume Lettre, Alexander P. Reine

**Blood-Cell Consortium Members**

*Authors from any of the three BCX1 articles published in the 2016 July issue of the American Journal of Human Genetics (PMID: 27346685, 27346686, 27346689).*

Abbas Dehghan, Akihiro Nomura, Alan B. Zonderman, Albert Hofman, Albert Vernon Smith, Alexander P. Reiner, Alexander Teumer, Allan Linneberg, Amber Burt, André G. Uitterlinden, Andreas Greinacher, Andres Metspalu, Andrew D. Johnson, Andrew J. Slater, Ani Manichaikul, Anne-Claire Vergnaud, Astrid Petersmann, Ayush Giri, Betina H. Thuesen, Bruce M. Psaty, Caroline Hayward, Caterina Vacchi-Suzzi, Chris J. O'Donnell, Christopher P. Cannon, Claudia Schurmann, Cornelia M. van Duijn, Cristen J. Willer, Dajiang J. Liu, David C. M. Liewald, David R. Crosslin, Dawn M. Waterworth, Deborah A. Nickerson, Diane M. Becker, Digna R. Velez Edwards, Emma Raitoharju, Eric Boerwinkle, Eric S. Torstenson, Erwin P. Bottinger, Ethan Lange, Eugene Braunwald, Evangelos Evangelou, Evelin Mihailov, Fernando Rivadeneira, Frank J. A. van Rooij, Frank Schmidt, Gina M. Peloso, Goncalo R. Abecasis, Guillaume Lettre, Gunnar Engström, Harvey D. White, He Gao, Henry Völzke, Heribert Schunkert, Ian J. Deary, Ioanna Tzoulaki, J.M. Starr, James G. Wilson, James S. Floyd, Jean-Claude Tardif, Jeanette Erdmann, Jennifer A. Brody, Jerome I. Rotter, Jette Bork-Jensen, Jin Li, Joel N. Hirschhorn, John D. Eicher, John D. Rioux, John M. Starr, Jussi Hernesniemi, Kenneth M. Rice, Kent D. Taylor, Kjell Nikus, Lars Wallentin, Latisha Love-Gregory, Laura M. Raffield, Lenore J. Launer, Leo-Pekka Lyytikäinen, Leslie A. Lange, Lewis C. Becker, Linda Polfus, Lisa R. Yanek, Marguerite R. Irvin, Marie-Pierre Dubé, Marju Orho-Melander, Mary Cushman, Melissa Richard, Michele K. Evans, Michelle L. O'Donoghue, Mika Kähönen, Mike A. Nalls, Ming-Huei Chen, Myriam Fornage, Nada A. Abumrad, Nathalie Chami, Nathan Pankratz, Nauder Faraday, Neil A. Zakai, Niels Grarup, Nilesh J. Samani, Nina Mononen, Olle Melander, Olli T. Raitakari, Oluf Pedersen, Oscar H. Franco, Panos Deloukas, Paul Elliott, Paul L. Auer, Raha Pazoki, Rakale Quarells, Rasika A. Mathias, Reedik Mägi, Russell P. Tracy, Ruth J.F. Loos, Salman M. Tajuddin, Samuel Lessard, Santhi K. Ganesh, Sekar Kathiresan, Simon de Denus, Stephen S. Rich, Tamara B. Harris, Terho Lehtimäki, Thomas Thiele, Tim Kacprowski, Todd L. Edwards, Tõnu Esko, Torben Hansen, Traci M. Bartz, Ursula M. Schick, Uwe Völker, Vilmundur Gudnason, W. David Hill, Yongmei Liu

**Additional acknowledgments and/or funding information**

**Montreal Heart Institute Biobank**

We thank all participants and staff of the André and France Desmarais Montreal Heart Institute’s (MHI) Biobank. The genotyping of the MHI Biobank was done at the MHI Pharmacogenomic Centre and funded by the MHI Foundation.

**BioMe**

The Mount Sinai IPM Biobank Program is supported by The Andrea and Charles Bronfman Philanthropies.

**Health2006/2008**

The Health2006 was financially supported by grants from the Velux Foundation; The Danish Medical Research Council, Danish Agency for Science, Technology and Innovation; The Aase and Ejner Danielsens Foundation; ALK-Abello A/S, Hørsholm, Denmark, and Research Centre for Prevention and Health, the Capital Region of Denmark. The Novo Nordisk Foundation Center for Basic Metabolic Research is an independent Research Center at the University of Copenhagen partially funded by an unrestricted donation from the Novo Nordisk Foundation (www.metabol.ku.dk). This work was supported by the Timber Merchant Vilhelm Bang’s Foundation, the Danish Heart Foundation (Grant number 07-10-R61-A1754-B838-22392F), and the Health Insurance Foundation (Helsefonden) (Grant number 2012B233).

**GeneSTAR**

GeneSTAR was supported by the National Institutes of Health/National Heart, Lung, and Blood Institute (U01 HL72518, HL087698, and HL112064) and by a grant from the National Institutes of Health/National Center for Research Resources (M01-RR000052) to the Johns Hopkins General Clinical Research Center. Genotyping services were provided through the RS&G Service by the Northwest Genomics Center at the University of Washington, Department of Genome Sciences, under U.S. Federal Government contract number HHSN268201100037C from the National Heart, Lung, and Blood Institute.

**BIOVU**

The dataset used in the analyses described were obtained from Vanderbilt University Medical Center's BioVU which is supported by institutional funding and by the Vanderbilt CTSA grant UL1 TR000445 from NCATS/NIH. Genome-wide genotyping was funded by NIH grants RC2GM092618 from NIGMS/OD and U01HG004603 from NHGRI/NIGMS. Funding for TLE and DRVE was provided by 1R21HL12142902 from NHLBI/NIH. Funding for the BioVU replication cohort was provided by 5R01HD074711 from NICHD/NIH.

**MESA**

MESA and the MESA SHARe project are conducted and supported by the National Heart, Lung, and Blood Institute (NHLBI) in collaboration with MESA investigators. Support for MESA is provided by contracts N01-HC-95159, N01-HC-95160, N01-HC-95161, N01-HC-95162, N01-HC-95163, N01-HC-95164, N01-HC-95165, N01-HC-95166, N01-HC-95167, N01-HC-95168, N01-HC-95169, UL1-TR-001079, UL1-TR-000040, and DK063491. MESA Family is conducted and supported by the National Heart, Lung, and Blood Institute (NHLBI) in collaboration with MESA investigators. Support is provided by grants and contracts R01HL071051, R01HL071205, R01HL071250, R01HL071251, R01HL071258, R01HL071259, by the National Center for Research Resources, Grant UL1RR033176, and the National Center for Advancing Translational Sciences, Grant UL1TR000124. Funding support for the inflammation dataset was provided by grant HL077449. The MESA Epigenomics & Transcriptomics Study was funded by NIA grant 1R01HL101250-01 to Wake Forest University Health Sciences.

**ARIC**

The Atherosclerosis Risk in Communities (ARIC) Study is carried out as a collaborative study supported by National Heart, Lung, and Blood Institute contracts (HHSN268201100005C, HHSN268201100006C, HHSN268201100007C, HHSN268201100008C, HHSN268201100009C, HHSN268201100010C, HHSN268201100011C, and HHSN268201100012C), R01HL087641, R01HL59367 and R01HL086694; National Human Genome Research Institute contract U01HG004402; and National Institutes of Health contract HHSN268200625226C. Infrastructure was partly supported by Grant Number UL1RR025005, a component of the National Institutes of Health and NIH Roadmap for Medical Research. The meta-analysis and meta-regression analyses were funded by grant R01 HL086694 from the National Heart, Lung, and Blood Institute.

**JHS**

The JHS is supported by contracts HHSN268201300046C, HHSN268201300047C, HHSN268201300048C, HHSN268201300049C, HHSN268201300050C from the National Heart, Lung, and Blood Institute and the National Institute on Minority Health and Health Disparities.

**WHI**

The WHI program is funded by the National Heart, Lung, and Blood Institute, the US National Institutes of Health and the US Department of Health and Human Services (HHSN268201100046C, HHSN268201100001C, HHSN268201100002C, HHSN268201100003C, HHSN268201100004C and HHSN271201100004C). Exome chip data and analysis were supported through the Exome Sequencing Project (NHLBI RC2 HL-102924, RC2 HL-102925 and RC2 HL-102926), the Genetics and Epidemiology of Colorectal Cancer Consortium (NCI CA137088), and the Genomics and Randomized Trials Network (NHGRI U01-HG005152).

**HANDLS**

The Healthy Aging in Neighborhoods of Diversity across the Life Span Study (HANDLS) research was supported by the Intramural Research Program of the NIH, National Institute on Aging and the National Center on Minority Health and Health Disparities (project # Z01-AG000513 and human subjects protocol # 2009-149). Data analyses for the HANDLS study utilized the computational resources of the NIH HPC Biowulf cluster at the National Institutes of Health, Bethesda, MD (http://hpc.nih.gov).

**GSK-STABILITY and SOLID TIMI-52**

The GSK-STABILITY and SOLID TIMI-52 studies were funded by GlaxoSmithKline.

**CHS**

This CHS research was supported by NHLBI contracts HHSN268201200036C, HHSN268200800007C, N01HC55222, N01HC85079, N01HC85080, N01HC85081, N01HC85082, N01HC85083, N01HC85086; and NHLBI grants HL080295, HL087652, HL103612, HL105756, HL120393, HL130114 with additional contribution from the National Institute of Neurological Disorders and Stroke (NINDS). Additional support was provided through AG023629 from the National Institute on Aging (NIA). The provision of genotyping data was supported in part by the National Center for Advancing Translational Sciences, CTSI grant UL1TR000124, and the National Institute of Diabetes and Digestive and Kidney Disease Diabetes Research Center (DRC) grant DK063491 to the Southern California Diabetes Endocrinology Research Center.

**Framingham Heart Study**

Genotyping, quality control and calling of the Illumina HumanExome BeadChip in the Framingham Heart Study was supported by funding from the National Heart, Lung and Blood Institute Division of Intramural Research (Daniel Levy and Christopher J. O’Donnell, Principal Investigators). Support for the centralized genotype calling was provided by Building on GWAS for NHLBI-diseases: the U.S. CHARGE consortium through the National Institutes of Health (NIH) American Recovery and Reinvestment Act of 2009 (5RC2HL102419). The NHLBI’s Framingham Heart Study is a joint project of the National Institutes of Health and Boston University School of Medicine and was supported by contract N01-HC-25195.

**SHIP and SHIP-TREND**

SHIP is part of the Community Medicine Research net of the University of Greifswald, Germany, which is funded by the Federal Ministry of Education and Research (grants no. 01ZZ9603, 01ZZ0103, and 01ZZ0403), the Ministry of Cultural Affairs as well as the Social Ministry of the Federal State of Mecklenburg-West Pomerania, and the network ‘Greifswald Approach to Individualized Medicine (GANI_MED)’ funded by the Federal Ministry of Education and Research (grant 03IS2061A). ExomeChip data have been supported by the Federal Ministry of Education and Research (grant no. 03Z1CN22) and the Federal State of Mecklenburg-West Pomerania.

**NWIGM**

This phase of the eMERGE Network was initiated and funded by the NHGRI through the following grants: U01HG8657 (Group Health Cooperative/University of Washington); U01HG8685 (Brigham and Women’s Hospital); U01HG8672 (Vanderbilt University Medical Center); U01HG8666 (Cincinnati Children’s Hospital Medical Center); U01HG6379 (Mayo Clinic); U01HG8679 (Geisinger Clinic); U01HG8680 (Columbia University Health Sciences); U01HG8684 (Children’s Hospital of Philadelphia); U01HG8673 (Northwestern University); U01HG8701 (Vanderbilt University Medical Center serving as the Coordinating Center); U01HG8676 (Partners Healthcare/Broad Institute); and U01HG8664 (Baylor College of Medicine). NWIGM dataset please also add "Additional support was provided by the University of Washington’s Northwest Institute of Genetic Medicine from Washington State Life Sciences Discovery funds (Grant 265508).

**FINCAVAS**

This work was supported by the Competitive Research Funding of the Tampere University Hospital (Grant 9M048 and 9N035), the Finnish Cultural Foundation, the Finnish Foundation for Cardiovascular Research, the Emil Aaltonen Foundation, Finland, and the Tampere Tuberculosis Foundation.

**YFS**

The Young Finns Study has been financially supported by the Academy of Finland: grants 286284, 134309 (Eye), 126925, 121584, 124282, 129378 (Salve), 117787 (Gendi), and 41071 (Skidi); the Social Insurance Institution of Finland; Kuopio, Tampere and Turku University Hospital Medical Funds (grant X51001); Juho Vainio Foundation; Paavo Nurmi Foundation; Finnish Foundation of Cardiovascular Research ; Finnish Cultural Foundation; Tampere Tuberculosis Foundation ; Emil Aaltonen Foundation ; and Yrjö Jahnsson Foundation.

**CARDIA**

The CARDIA Study is conducted and supported by the National Heart, Lung, and Blood Institute in collaboration with the University of Alabama at Birmingham (HHSN268201300025C & HHSN268201300026C), Northwestern University (HHSN268201300027C), University of Minnesota (HHSN268201300028C), Kaiser Foundation Research Institute (HHSN268201300029C), and Johns Hopkins University School of Medicine (HHSN268200900041C). CARDIA is also partially supported by the Intramural Research Program of the National Institute on Aging. Exome Chip genotyping was supported from grants R01-HL093029 and U01- HG004729 to MF. This manuscript has been reviewed and approved by CARDIA for scientific content.

**HABC**

HABC funding/acknowledgement: The Health ABC Study was supported by NIA contracts N01AG62101, N01AG62103, and N01AG62106 and, in part, by the NIA Intramural Research Program. The genome-wide association study was funded by NIA grant 1R01AG032098-01A1 to Wake Forest University Health Sciences and genotyping services were provided by the Center for Inherited Disease Research (CIDR). CIDR is fully funded through a federal contract from the National Institutes of Health to The Johns Hopkins University, contract number HHSN268200782096C. This study utilized the high-performance computational capabilities of the Biowulf Linux cluster at the National Institutes of Health, Bethesda, Md. (<http://biowulf.nih.gov>).

**EGCUT**

This study was supported by EU H2020 grants 692145, 676550, 654248, Estonian Research Council Grant IUT20-60, NIASC, EIT – Health and NIH-BMI grant 2R01DK075787-06A1.

**AIRWAVE**

The Airwave Study is funded by the Home Office (grant number 780-TETRA) with additional support from the National Institute for Health Research (NIHR) Imperial College Healthcare NHS Trust (ICHNT) and Imperial College Biomedical Research Centre (BRC). Paul Elliott is supported by the ICHNT and Imperial College BRC, the MRC-PHE Centre for Environment and Health (MR/L01341X/1) and the NIHR Health Protection Research Unit on Health Impact of Environmental Hazards (HPRU-2012-10141). This work used the computing resources of the UK MEDical BIOinformatics partnership (UK MED-BIO) supported by the Medical Research Council (MR/L01632X/1).
