# Supplementary material for: Rare coding variants pinpoint genes that control human hematological traits
Source: PLoS Genet. 2017 Aug 7;13(8):e1006925. doi: 10.1371/journal.pgen.1006925 (PMC5560754; doi:10.1371/journal.pgen.1006925)
Supplement: S1 Table — Chromosomes and positions are on build hg19 of the human genome. The direction of the effect sizes (Beta) is for allele A2. Beta and standard errors (SE) are in standard deviation units. Mono, monocyte; WBC, white blood cell count; MCH, mean corpuscular hemoglobin; Lympho, lymphocyte; MPV, mean platelet volume; HGB, hemoglobin; RBC, red blood cell count; Baso, basophil. (DOCX) [file pgen.1006925.s002.docx]

**Table S1. Coding or splice site variants with a minor allele frequency <1% that were excluded during quality-control.** Chromosomes and positions are on build hg19 of the human genome. The direction of the effect sizes (Beta) is for allele A2. Beta and standard errors (SE) are in standard deviation units. Mono, monocyte; WBC, white blood cell count; MCH, mean corpuscular hemoglobin; Lympho, lymphocyte; MPV, mean platelet volume; HGB, hemoglobin; RBC, red blood cell count; Baso, basophil.

| **SNPID** | **Chr (Pos)** | **Gene** | **Annotation (VEP)** | **A1/A2** | **Freq (A2)** | **Trait** | **Beta** | **SE** | **PVAL** | **Exclusion criteria** |
| --- | --- | --- | --- | --- | --- | --- | --- | --- | --- | --- |
| rs185127229 | 1 (93676434) | *CCDC18* | missense | T/C | 0.9986 | Mono | -0.4605 | 0.0565 | 3.56E-16 | BCX and UKBB results are discordant |
| rs138821617 | 6 (26156752) | *HIST1H1E* | missense | T/C | 0.9999 | MCH | -2.436 | 0.3318 | 2.12E-13 | BCX and UKBB results are discordant |
| rs140451451 | 6 (30080441) | *TRIM31-AS1* | missense | A/G | 0.0016 | Lympho | -0.6384 | 0.0882 | 4.47E-13 | HLA region |
| rs144731721 | 6 (30124811) | *TRIM10* | missense | T/C | 0.9922 | RDW | -0.1244 | 0.0212 | 4.77E-09 | HLA region |
| rs141504131 | 6 (30166600) | *TRIM26* | missense | T/C | 0.9987 | Lympho | 1.0237 | 0.1256 | 3.64E-16 | BCX and UKBB results are discordant |
| rs142398523 | 6 (30520367) | *GNL1* | missense | T/C | 0.9933 | WBC | -0.1176 | 0.0192 | 8.39E-10 | HLA region |
| rs150614928 | 6 (30610758) | *ATAT1* | missense | T/G | 0.993 | WBC | -0.1083 | 0.0194 | 2.18E-08 | HLA region |
| rs200530869 | 6 (30671952) | *MDC1-AS1* | missense | T/C | 0.9969 | MPV | 0.5777 | 0.0657 | 1.44E-18 | BCX and UKBB results are discordant |
| rs2517560 | 6 (30680968) | *MDC1-AS1* | missense | T/C | 0.9917 | HGB | 0.0918 | 0.0166 | 3.26E-08 | HLA region |
| rs55787895 | 6 (30862440) | *DDR1* | missense | A/G | 0.0095 | Lympho | 0.0948 | 0.0168 | 1.77E-08 | HLA region |
| rs35595439 | 6 (31605016) | *PRRC2A* | missense | T/C | 0.0086 | Lympho | 0.1365 | 0.0178 | 1.82E-14 | HLA region |
| rs28399992 | 6 (31732059) | *MSH5-SAPCD1* | missense | T/C | 0.0069 | RBC | -0.1222 | 0.0207 | 3.75E-09 | HLA region |
| rs138258006 | 6 (31762624) | *VARS* | missense | A/G | 0.9978 | MPV | 0.688 | 0.0648 | 2.43E-26 | HLA region |
| rs45484591 | 6 (31918468) | *CFB* | missense | A/C | 0.0086 | Lympho | 0.1321 | 0.0186 | 1.24E-12 | HLA region |
| rs6910390 | 6 (32041621) | *TNXB* | missense | A/G | 0.993 | Mono | -0.135 | 0.0201 | 1.75E-11 | HLA region |
| rs138771398 | 6 (32063460) | *TNXB* | missense | A/G | 0.9971 | Lympho | -0.2132 | 0.0331 | 1.15E-10 | HLA region |
| rs80096349 | 6 (32151458) | *AGER* | missense | A/G | 0.9943 | Lympho | -0.1605 | 0.0226 | 1.26E-12 | HLA region |
| rs72846312 | 6 (32170247) | *NOTCH4* | missense | T/C | 0.9953 | WBC | 0.4122 | 0.0677 | 1.13E-09 | HLA region |
| rs150079294 | 6 (32180684) | *NOTCH4* | missense | A/C | 0.0065 | MPV | 0.207 | 0.0344 | 1.75E-09 | BCX and UKBB results are discordant |
| rs115021976 | 6 (37247114) | *TBC1D22B* | missense | T/C | 0.9987 | MCH | -0.3172 | 0.0504 | 3.13E-10 | HLA region |
| rs185829668 | 7 (6505869) | *KDELR2* | missense | T/G | 0.9969 | Baso | 0.4927 | 0.0892 | 3.31E-08 | Variant is missing in the BCX dataset |
| rs201806626 | 8 (132052282) | *ADCY8* | missense | T/C | 0.9968 | MCV | -0.7458 | 0.0943 | 2.66E-15 | Variant is missing in the BCX dataset |
| rs189563213 | 10 (8006547) | *TAF3* | missense | C/G | 1 | WBC | -0.9498 | 0.1118 | 1.95E-17 | BCX and UKBB results are discordant |
| rs33971440 | 11 (5248159) | *HBB* | splice_donor | T/C | 0.9999 | MCV | 2.0295 | 0.2463 | 1.73E-16 | BCX and UKBB results are discordant |
| rs144349650 | 11 (55432976) | *OR4C6* | missense | C/G | 0 | WBC | 0.7008 | 0.1061 | 4.02E-11 | BCX and UKBB results are discordant |
