# Supplementary material for: Rare coding variants pinpoint genes that control human hematological traits
Source: PLoS Genet. 2017 Aug 7;13(8):e1006925. doi: 10.1371/journal.pgen.1006925 (PMC5560754; doi:10.1371/journal.pgen.1006925)
Supplement: S2 Table — Chromosomes and positions are on build hg19 of the human genome. The direction of the effect sizes (Beta) is for allele A2. Beta and standard errors (SE) are in standard deviation units. MPV, mean platelet volume; MCH, mean corpuscular hemoglobin; RDW, red blood cell distribution width; PLT, platelet count; WBC, white blood cell count; MCHC, mean corpuscular hemoglobin concentration; Mono, monocyte; Neutro, neutrophil; HGB, hemoglobin. (DOCX) [file pgen.1006925.s003.docx]

**Table S2. Coding or splice site variants with a minor allele frequency <1% that were previously reported to associate with blood-cell traits.** Chromosomes and positions are on build hg19 of the human genome. The direction of the effect sizes (Beta) is for allele A2. Beta and standard errors (SE) are in standard deviation units. MPV, mean platelet volume; MCH, mean corpuscular hemoglobin; RDW, red blood cell distribution width; PLT, platelet count; WBC, white blood cell count; MCHC, mean corpuscular hemoglobin concentration; Mono, monocyte; Neutro, neutrophil; HGB, hemoglobin.

| **SNPID** | **Chr (Pos)** | **Gene** | **Annotation (VEP)** | **A1/A2** | **Freq (A2)** | **Trait** | **Beta** | **SE** | **PVAL** | **References** |
| --- | --- | --- | --- | --- | --- | --- | --- | --- | --- | --- |
| rs34338164 | 2 (68615546) | *PLEK* | missense | A/C | 0.0093 | MPV | -0.101 | 0.0166 | 1.21E-09 | Astle et al., Cell, 2016 |
| rs76036957 | 2 (113496453) | *CKAP2L* | missense | A/G | 0.9907 | MPV | 0.1941 | 0.0168 | 7.16E-31 | Astle et al., Cell, 2016 |
| rs56407180 | 3 (124303696) | *KALRN* | stop_gained | T/C | 0.9977 | MPV | 0.4902 | 0.0488 | 1.05E-23 | Astle et al., Cell, 2016 |
| rs8177318 | 3 (133467377) | *TF* | missense | A/T | 0.9987 | MCH | 0.4602 | 0.046 | 1.57E-23 | Astle et al., Cell, 2016 |
| rs150854910 | 3 (133476769) | *TF* | missense | T/C | 0.9987 | MCH | -0.3383 | 0.0426 | 1.95E-15 | Astle et al., Cell, 2016 |
| rs143845082 | 3 (171417570) | *PLD1* | missense | A/G | 0.9956 | RDW | -0.2012 | 0.0249 | 6.70E-16 | Astle et al., Cell, 2016 |
| rs149535568 | 3 (171442535) | *PLD1* | missense | A/C | 0.9959 | RDW | -0.2181 | 0.0287 | 2.76E-14 | Astle et al., Cell, 2016 |
| rs146771786 | 6 (16286061) | *GMPR* | missense | A/C | 0.9982 | MCH | -0.2916 | 0.0438 | 2.86E-11 | Astle et al., Cell, 2016 |
| rs150221602 | 9 (5081828) | *JAK2* | missense | C/G | 0.999 | PLT | -0.4634 | 0.047 | 5.82E-23 | Astle et al., Cell, 2016 |
| rs41316003 | 9 (5126343) | *JAK2* | missense | A/G | 0.9948 | PLT | -0.1614 | 0.0214 | 4.88E-14 | Astle et al., Cell, 2016 |
| rs143034248 | 11 (118081270) | *JAML* | missense | T/C | 0.9942 | WBC | -0.1688 | 0.0188 | 2.52E-19 | Astle et al., Cell, 2016 |
| rs202099525 | 16 (88788037) | *PIEZO1* | missense | A/G | 0.9993 | MCHC | 0.3779 | 0.0507 | 9.23E-14 | Astle et al., Cell, 2016 |
| rs144120533 | 17 (76134467) | *TMC8* | missense | T/C | 0.9964 | RDW | 0.1832 | 0.0292 | 3.42E-10 | Astle et al., Cell, 2016 |
| rs140221307 | 22 (17586757) | *IL17RA* | missense | T/C | 0.0052 | Mono | -0.5662 | 0.0237 | 2.00E-126 | Astle et al., Cell, 2016 |
| rs55799208 | 2 (218999982) | *CXCR2* | missense | A/G | 0.9973 | Neutro | 0.3893 | 0.0308 | 9.83E-37 | Auer et al., Nat Genet, 2014 |
| rs61733609 | 2 (219000267) | *CXCR2* | missense | A/G | 0.9985 | WBC | 0.4775 | 0.0506 | 3.60E-21 | Auer et al., Nat Genet, 2014 |
| rs139178017 | 7 (100225847) | *TFR2* | splice_region | T/C | 0.9951 | HGB | -0.1921 | 0.0211 | 8.18E-20 | Auer et al., Nat Genet, 2014 |
| rs62483572 | 7 (100319633) | *EPO* | missense | A/G | 0.9952 | HGB | 0.227 | 0.0223 | 2.22E-24 | Auer et al., Nat Genet, 2014 |
| rs148636776 | 12 (111885295) | *SH2B3* | missense | A/G | 0.9995 | PLT | -0.5746 | 0.0614 | 8.08E-21 | Auer et al., Nat Genet, 2014 |
| rs72650673 | 12 (111885310) | *SH2B3* | missense | A/G | 0.9985 | PLT | -0.4546 | 0.0397 | 2.09E-30 | Auer et al., Nat Genet, 2014 |
| rs41303899 | 20 (57598808) | *TUBB1* | missense | A/G | 0.9985 | MPV | -0.7776 | 0.0428 | 8.49E-74 | Auer et al., Nat Genet, 2014 |
| rs116100695 | 1 (155261709) | *PKLR* | missense | A/G | 0.9972 | HGB | 0.1832 | 0.0285 | 1.33E-10 | Chami et al., AJHG, 2016 |
| rs34968964 | 5 (75960968) | *IQGAP2* | missense | C/G | 0.9946 | MPV | -0.3097 | 0.0225 | 3.58E-43 | Eicher et al., AJHG, 2016 |
| rs3746072 | 19 (3179884) | *S1PR4* | missense | T/G | 0.993 | WBC | 0.2528 | 0.0189 | 7.50E-41 | Pankratz et al., Nat Genet, 2016 |
| rs149771513 | 22 (17588658) | *IL17RA* | missense | A/G | 0.9993 | Mono | -0.869 | 0.0851 | 1.75E-24 | Pankratz et al., Nat Genet, 2016 |
