# Supplementary material for: Rare coding variants pinpoint genes that control human hematological traits
Source: PLoS Genet. 2017 Aug 7;13(8):e1006925. doi: 10.1371/journal.pgen.1006925 (PMC5560754; doi:10.1371/journal.pgen.1006925)
Supplement: S6 Table — When Pcond >0.002 (Bonferroni correction for 31 variants tested), we performed pairwise conditional analyses with markers at the locus to identify the variant that account for the association signal. This variant is listed in the Tagging variant column, along with its minor allele frequency and functional annotation. MPV, mean platelet volume; MCH, mean corpuscular hemoglobin; RDW, red blood cell distribution width; PLT, platelet count; WBC, white blood cell count; MCHC, mean corpuscular hemoglobin concentration; Mono, monocyte; Neutro, neutrophil; HGB, hemoglobin; MCV, mean corpuscular volume; Eosin, eosinophil; RBC, red blood cell count; HCT, hematocrit. (DOCX) [file pgen.1006925.s007.docx]

**Table S6. Conditional results in the UK Biobank (UKBB).** When *P*_cond_ >0.002 (Bonferroni correction for 31 variants tested), we performed pairwise conditional analyses with markers at the locus to identify the variant that account for the association signal. This variant is listed in the Tagging variant column, along with its minor allele frequency and functional annotation. MPV, mean platelet volume; MCH, mean corpuscular hemoglobin; RDW, red blood cell distribution width; PLT, platelet count; WBC, white blood cell count; MCHC, mean corpuscular hemoglobin concentration; Mono, monocyte; Neutro, neutrophil; HGB, hemoglobin; MCV, mean corpuscular volume; Eosin, eosinophil; RBC, red blood cell count; HCT, hematocrit.

| **SNPID** | **CHR  (POS)** | **Gene** | **Main phenotype** | **UKBB *P*_non-cond_** | **UKBB *P*_cond_** | **Tagging variant** | **Minor allele frequency in UKBB (allele)** | **Gene** | **Annotation** | **Comment** |
| --- | --- | --- | --- | --- | --- | --- | --- | --- | --- | --- |
| rs148916169 | 1 (36932463) | *CSF3R* | WBC | 6.09E-07 | 0.61 | 1:36947888_G_A (rs3917914) | 0.011455 (A) | *CSF3R* | intronic |  |
| rs138903557 | 2 (24245713) | *MFSD2B* | MCV | 2.70E-07 | 0.0000322 | - | - | - | - |  |
| rs147820690 | 2 (160735174) | *LY75-CD302* | PLT | 0.0043 | 0.000694 | - | - | - | - |  |
| rs116274727 | 2 (192701265) | *SDPR* | MPV | 7.77E-08 | 9.57E-12 | - | - | - | - |  |
| rs28910273 | 3 (142188337) | *ATR* | MCV | 1.70E-08 | 3.74E-10 | - | - | - | - |  |
| rs77208665 | 3 (142274770) | *ATR* | MCV | 1.38E-05 | 5.16E-07 | - | - | - | - |  |
| rs151053159 | 5 (1078832) | *SLC12A7* | RDW | 9.81E-10 | 0.001195 | - | - | - | - |  |
| rs121434346 | 5 (1212453) | *SLC6A19* | RDW | 2.06E-05 | 0.63 | 5:1058387_G_A (rs111910553) | 0.012142 (A) | *SLC12A7* | intronic |  |
| rs145535174 | 6 (161134124) | *PLG* | PLT | 0.00017 | 3.62E-05 | - | - | - | - |  |
| rs74848966 | 7 (100365467) | *ZAN* | MCH | 2.20E-06 | 0.27 | 7:100414147_G_A (rs116979562) | 0.028035 (A) | *EPHB4* | intronic | This association with MCH is independent of genotypes at a rare missense variant in *EPO* (rs62483572) and a rare splice site variant in *TFR2* (rs139178017) (Auer et *al., Nature Genet*., 2014). |
| rs141547371 | 9 (214606) | *C9orf66* | MPV | 5.12E-08 | 0.0115 | 9:329343_CA_C (rs56318916) | 0.194322 (C) | *DOCK8* | intronic |  |
| rs146597587 | 9 (6255967) | *IL33* | Eosin | 1.02E-17 | 0.88 | 9:6288604_G_C (rs145697747) | 0.004812 (C) | - | intergenic |  |
| rs146879704 | 9 (114886569) | *SUSD1* | HGB | 1.87E-06 | 3.00E-05 | - | - | - | - |  |
| rs141547732 | 9 (136280025) | *REXO4* | RBC | 8.05E-07 | 0.40 | 9:136130855_TTCTG_T (rs149037075) | 0.061437 (T) | *ABO* | 3'UTR |  |
| rs71508957 | 10 (64927837) | *JMJD1C* | MPV | 4.14E-08 | 0.0298 | 10:65066186_G_T (rs10761741) | 0.415626 (T) | *JMJD1C* | intronic |  |
| rs61748606 | 11 (230474) | *SIRT3* | MPV | 3.74E-10 | 0.46 | 11:196944_C_T (rs11604127) | 0.236721 (T) | *ODF3* | 5'UTR |  |
| rs138326449 | 11 (116701354) | *APOC3* | RDW | 4.48E-08 | 2.72E-09 | - | - | - | - |  |
| rs150349412 | 12 (112184086) | *ACAD10* | PLT | 2.19E-06 | 0.65 | 12:112248761_A_G (rs530203431) | 0.001185 (G) | *-* | intergenic | This association with PLT count is independent from genotypes at 3 rare missense variants in *SH2B3* (rs3184504, rs148636776, rs72650673)(Auer et *al., Nature Genet*., 2014). |
| rs145120027 | 12 (122439451) | *WDR66* | MPV | 2.02E-09 | 0.84 | 12:122216910_A_G (rs11553699) | 0.139106 (G) | *RHOF, TMEM120B* | 3'UTR, 5'UTR |  |
| rs17881033 | 12 (122763670) | *CLIP1* | MPV | 2.44E-20 | 0.66 | 12:122216910_A_G (rs11553699) | 0.139106 (G) | *RHOF, TMEM120B* | 3'UTR, 5'UTR |  |
| rs151322438 | 12 (123335398) | *HIP1R* | MPV | 1.21E-13 | 0.20 | 12:122216910_A_G (rs11553699) | 0.139106 (G) | *RHOF, TMEM120B* | 3'UTR, 5'UTR |  |
| rs146030737 | 13 (28626716) | *FLT3* | Mono | 3.81E-06 | 0.52 | 13:28649996_C_A (rs189992044) | 0.003616 (A) | *FLT3* | intronic |  |
| rs182782800 | 13 (73319139) | *BORA* | MCV | 3.26E-09 | 0.124 | 13:73275607_T_C (rs184739375) | 0.0042967 (C) | *-* | intergenic |  |
| rs138887682 | 14 (103568488) | *EXOC3L4* | MPV | 6.37E-07 | 1.36E-10 | - | - | - | - |  |
| rs148718670 | 14 (103574815) | *EXOC3L4* | MPV | 4.24E-06 | 3.39E-03 | 14:103566835_C_G (rs2297066) | 0.242337 (G) | *EXOC3L4* | missense_variant |  |
| rs184575290 | 15 (80191280) | *ST20* | Mono | 1.57E-10 | 9.30E-09 | - | - | - | - |  |
| rs57268939 | 16 (319547) | *FAM234A* | MCH | 6.05E-11 | 1.85E-09 | - | - | - | - |  |
| rs147810715 | 16 (30999491) | *HSD3B7* | MCH | 3.22E-08 | 4.74E-08 | - | - | - | - |  |
| rs35266519 | 17 (38062390) | *GSDMB* | Neutro | 3.18E-07 | 0.32 | 17:38173637_C_T (rs146890554) | 0.012946 (T) | *CSF3* | 3'UTR |  |
| rs150420714 | 19 (50017538) | *FCGRT* | HCT | 9.77E-05 | 2.76E-05 | - | - | - | - |  |
| rs201148397 | 22 (37482458) | *TMPRSS6* | MCH | 3.89E-09 | 8.86E-05 | - | - | - | - |  |
