# Supplementary material for: Rare coding variants pinpoint genes that control human hematological traits
Source: PLoS Genet. 2017 Aug 7;13(8):e1006925. doi: 10.1371/journal.pgen.1006925 (PMC5560754; doi:10.1371/journal.pgen.1006925)
Supplement: S7 Table — All analyses were corrected for age and sex. UKBB, UK Biobank; MHIBB, Montreal Heart Institute Biobank; WHI, Women’s Health Initiative. The direction of the odds ratios is for the rare G-allele at rs145535174 (allele A2). (DOCX) [file pgen.1006925.s008.docx]

**Table S7. Association between *PLG*-rs145535174 and thrombotic events.** All analyses were corrected for age and sex. UKBB, UK Biobank; MHIBB, Montreal Heart Institute Biobank; WHI, Women’s Health Initiative. The direction of the odds ratios is for the rare G-allele at rs145535174 (allele A2).

| **Disease** | **Study** | **N_cases_** | **N_ctrls_** | **A1/A2** | **Freq (A2)** | **Odds ratio** | **95% confidence interval** | ***P*-value** |
| --- | --- | --- | --- | --- | --- | --- | --- | --- |
| Myocardial infarction | UKBB | 3,689 | 85,783 | A/G | 0.0006 | 0.76 | 0.20 – 2.03 | 0.63 |
| Stroke | UKBB | 2,640 | 92,533 | A/G | 0.0006 | 1.17 | 0.35 – 2.89 | 0.76 |
| Venous thromboembolism | UKBB | 708 | 135,376 | A/G | 0.0006 | 4.01 | 1.00 – 10.68 | 0.0087 |
|  | MHIBB | 487 | 10,350 | A/G | 0.0012 | 0.86 | 0.12 – 6.29 | 0.88 |
|  | WHI | 1,134 | 24,604 | A/G | 0.00093 | 0.998 | 0.134- 0.742 | 0.998 |
