# Supplementary material for: Rare coding variants pinpoint genes that control human hematological traits
Source: PLoS Genet. 2017 Aug 7;13(8):e1006925. doi: 10.1371/journal.pgen.1006925 (PMC5560754; doi:10.1371/journal.pgen.1006925)
Supplement: S8 Table — For each of the 31 novel rare coding or splice site variants presented in this study, we queried the corresponding loci in previous GWAS of blood-cell traits and highlighted previously prioritized candidate genes based on functional annotation (missense, splice site) or regulatory (eQTL) effect in the GTEx database. (DOCX) [file pgen.1006925.s009.docx]

**Table S8. Prioritization of candidate genes.** For each of the 31 novel rare coding or splice site variants presented in this study, we queried the corresponding loci in previous GWAS of blood-cell traits and highlighted previously prioritized candidate genes based on functional annotation (missense, splice site) or regulatory (eQTL) effect in the GTEx database.

| **SNPID** | **CHR (POS)** | **Gene** | **Phenotype** | **Coding or splice site variants** | **eQTLs in GTEx** | **Comments** |
| --- | --- | --- | --- | --- | --- | --- |
| rs148916169 | 1 (36932463) | *CSF3R* | WBC | No coding/splice site variants | No eQTLS. | No candidates. |
| rs138903557 | 2 (24245713) | *MFSD2B* | MCV | No coding/splice site variants | rs925228 and rs7561675 are eQTLs for TP53I3, FKBP1B, UBXN2A, PFN4, and FAM228B. | These genes have no links to hematopoiesis. |
| rs147820690 | 2 (160735174) | *LY75-CD302* | PLT | Two low-frequency (rs148440689, missense_MARCH7; rs78446341, missense_LY75_CD302) variants. | rs12052715 is an eQTL for LY75 and WDSUB1. rs76840964 is an eQTL for LY75 and PLA2R1. | LY75-CD302 is already discussed in the manuscript. Other genes have no known links to hematopoiesis. |
| rs116274727 | 2 (192701265) | *SDPR* | MPV | No coding/splice site variants | No eQTLS. | No candidates |
| rs28910273 | 3 (142188337) | *ATR* | MCV | No coding/splice site variants | rs2227930, rs7428496, and rs1357529 are eQTLs for XRN1, ATR, and RP11-485G4.2. rs9820353 is an eQTL for XRN1 and ATR. | ATR is already discussed in the manuscript. XRN1 has no known links to hematopoiesis. |
| rs77208665 | 3 (142274770) | *ATR* | MCV |  |  |  |
| rs151053159 | 5 (1078832) | *SLC12A7* | RDW | No coding/splice site variants | rs6883412 is an eQTL for CTD-2228K2.7. rs35188965 and rs13181874 are eQTLs for SLC12A7. | SLC12A7 is already discussed in the manuscript. CTD-2228K2.7 is an RNA gene of unknown functions. |
| rs121434346 | 5 (1212453) | *SLC6A19* | RDW | No coding/splice site variants | rs6883412 is an eQTL for CTD-2228K2.7. rs35188965 and rs13181874 are eQTLs for SLC12A7. | SLC12A7 is already discussed in the manuscript. CTD-2228K2.7 is an RNA gene of unknown functions. |
| rs145535174 | 6 (161134124) | *PLG* | PLT | Novel locus | | |
| rs74848966 | 7 (100365467) | *ZAN* | MCH | One low-frequency (rs41295942, missense_TRF2) and three rare (rs139178017, splice_site_TRF2; rs62482253, splice_site_GNB2; rs62483572, missense_EPO) variants. | rs41295942 is an eQTL for ZSCAN21 and AP4M1. rs4548095 is an eQTL for TFR2, MOSPD3, and EPO. rs7385804, rs2075672 and rs9801017 are eQTLs for TFR2, MOSPD3, EPO, and PCOLCE-AS1. rs4434553 is an eQTL for MOSPD3, PCOLCE-AS1, and STAG3L5P. rs11772705 is an eQTL for ACHE, MOSPD3, EPO, and GIGYF1. rs551238 is an eQTL for MOSPD3, PILRB, EPO, ZAN, and PCOLCE-AS1. rs116979562 is an eQTL for AP4M1. | TRF2 and EPO are well-known regulators of erythropoiesis. Other genes cannot be linked to hematopoiesis given current knowledge. |
| rs141547371 | 9 (214606) | *C9orf66* | MPV | No coding/splice site variants | rs471756 and rs785830 are eQTL for DOCK8 and CBWD1. rs2992836 is an eQTL for DOCK8. | DOCK8 mutations cause immunodeficiency, although the link with platelet is not immediately obvious. |
| rs146597587 | 9 (6255967) | *IL33* | Eosin | No coding/splice site variants | No eQTLS. | No candidates |
| rs146879704 | 9 (114886569) | *SUSD1* | HGB | No coding/splice site variants | No eQTLS. | No candidates |
| rs141547732 | 9 (136280025) | *REXO4* | RBC | No coding/splice site variants | rs10901252, rs8176759, rs8176747, rs8176671, and rs8176644 are an eQTL for OBP2B and ABO. rs687289 is an eQTL for ABO. rs9411378 is an eQTL for ABO and RP11-430N14.4. rs635634 is an eQTL for SURF1, ABO, and RP11-430N14.4 | ABO encodes the glycosyltransferase responsible for blood group. |
| rs71508957 | 10 (64927837) | *JMJD1C* | MPV | One low-frequency (rs41274072, missense_JMJD1C) | rs41274072 is an eQTL for REEP3. rs10995477 is an eQTL for NRBF2. rs10761731, rs10761741, rs10740118, and rs7896518 are eQTL for NRBF2 and MRPL35P2. | JMJD1C has been linked to acute myeloid leukemia, although link to platelet is not obvious. |
| rs61748606 | 11 (230474) | *SIRT3* | MPV | No coding/splice site variants | rs11604127 and rs55966801 are eQTL for SIRT3, NLRP6, BET1L, and RIC8A. | Sirtuins are implicated in platelet aging. Other genes have no known links to hematopoiesis. |
| rs138326449 | 11 (116701354) | *APOC3* | RDW | No coding/splice site variants | rs2070667 is an eQTL for APOA1. | APOA1 has not been linked to hematopoiesis. |
| rs150349412 | 12 (112184086) | *ACAD10* | PLT | One common (rs3184504, missense_SH2B3) and one rare (rs148636776, missense_SH2B3) variants | rs3803170 is an eQTL of MAPKAPK5, ALDH2, HECTD4, and TMEM116. rs3184504, rs653178, and rs4766578 are eQTL of ALDH2 and HECTD4. rs615134 is an eQTL for BRAP, ALDH2, and ADAM1A. | SH2B3 is a key regulator of hematopoiesis (encodes LNK). |
| rs145120027 | 12 (122439451) | *WDR66* | MPV | No coding/splice site variants | rs11553699 is an eQTL for WDR66 and HPD. rs11043280 and rs7954584 are eQTL for PSMD9, BCL7A, WDR66 and HPD. rs116896792 is an eQTL for CDK2AP1. | Result is concordant for WDR66 (rare missense and common eQTL) but the gene itself has not been linked functionally to platelet biology. |
| rs17881033 | 12 (122763670) | *CLIP1* | MPV |  |  |  |
| rs151322438 | 12 (123335398) | *HIP1R* | MPV |  |  |  |
| rs146030737 | 13 (28626716) | *FLT3* | Mono | No coding/splice site variants | rs61946325 and rs2491244 are eQTL for FLT3. | FLT3 is critical for WBC differentiation. Also plays a role in leukemogenesis. |
| rs182782800 | 13 (73319139) | *BORA* | MCV | No coding/splice site variants | No eQTLS. | No candidates |
| rs13888768 | 14 (103568488) | *EXOC3L4* | MPV | Two common (14:103566835, missense_EXOC3L4; 14:103576444, missense_EXOC3L4) variants | No eQTLS. | Result is concordant for EXOC3L4 (common and rare missense) but the gene itself has no known functions. |
| rs148718670 | 14 (103574815) | *EXOC3L4* | MPV |  |  |  |
| rs184575290 | 15 (80191280) | *ST20* | Mono | No coding/splice site variants | rs2115536 is an eQTL for ST20 and MTHFS. | Results are concordant but the genes themselves have no known links to WBC biology. |
| rs57268939 | 16 (319547) | *FAM234A* | MCH | No coding/splice site variants | Complex regulatory pattern of expression for alpha-globin genes. | alpha-globin locus has previously been associated with RBC traits. |
| rs147810715 | 16 (30999491) | *HSD3B7* | MCH | No coding/splice site variants | rs1046276 and rs12928852 are eQTL for STX1B, HSD3B7, STX4, KAT8, RNF40, ITGAX, PRSS53, ZNF668, ZNF646, and VKORC1. rs8050500 is an eQTL for COX6A2, ITGAD, and ZNF843. rs62051538 is an eQTL for ZNF843. | ITGAX encodes an integrin at the surface of WBC. VKORC1 plays a role in the vitamin K pathway (coagulation). |
| rs35266519 | 17 (38062390) | *GSDMB* | Neutro | No coding/splice site variants | rs13313564 is an eQTL for GSDMB. rs921650 is and eQTL for GSDMB, GSDMA, and ORMDL3. | Whereas the rare missense variant support GSDMB, eQTL results highlight GSDMB/A and ORMDL3. |
| rs150420714 | 19 (50017538) | *FCGRT* | HCT | No coding/splice site variants | No eQTLS. | No candidates |
| rs201148397 | 22 (37482458) | *TMPRSS6* | MCH | One common (22:37462936, missense_TMPRSS6) variant | rs6000550 and rs2076085 is an eQTL for MPST. rs9619658 is an eQTL for TMPRSS6. | TMPRSS6 is a key regulator of iron metabolism and plays a critical role in RBC biology. |
