# Supplementary figures and images for: Rare coding variants pinpoint genes that control human hematological traits
Source: PLoS Genet. 2017 Aug 7;13(8):e1006925. doi: 10.1371/journal.pgen.1006925 (PMC5560754; doi:10.1371/journal.pgen.1006925)

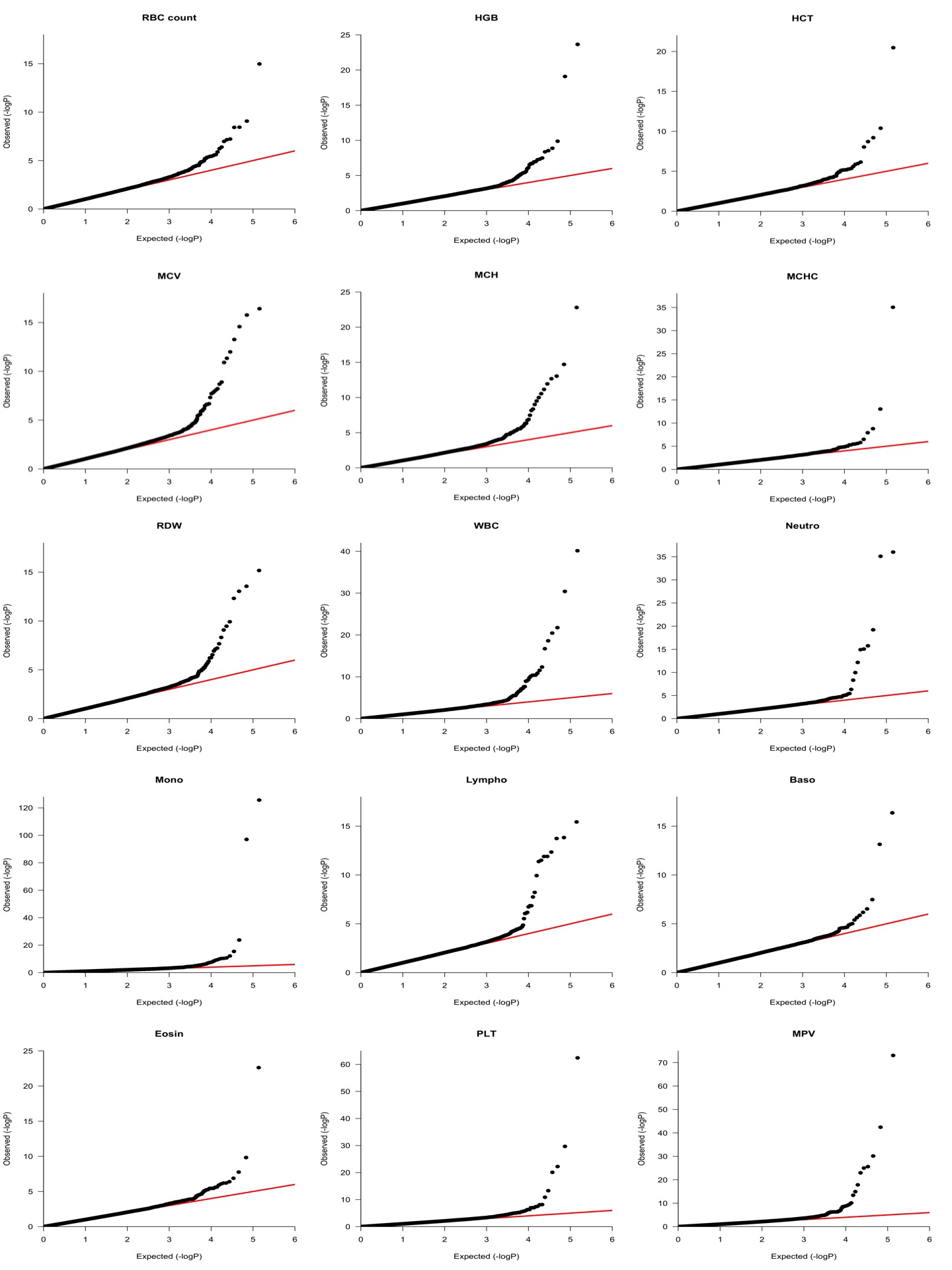

Supplement: S1 Fig — We present association results for 15 hematological traits analyzed at 137,086 variants in up to 308,572 participants. (JPG) [file pgen.1006925.s011.jpg]
